# Asthma and Food Allergy: Which Risks?

**DOI:** 10.3390/medicina55090509

**Published:** 2019-08-21

**Authors:** Emanuela di Palmo, Marcella Gallucci, Francesca Cipriani, Luca Bertelli, Arianna Giannetti, Giampaolo Ricci

**Affiliations:** Pediatric Unit, Department of Medical and Surgical Sciences, University of Bologna, 40138 Bologna, Italy

**Keywords:** asthma, atopic march, food allergy, anaphylaxis, children

## Abstract

Over the past few decades, an increase in the prevalence of asthma and food allergy has been observed in the pediatric population. In infants, food sensitization, particularly to egg, has increased the risk of developing allergic asthma. This is even more likely if sensitization to food allergens occurs early within the first few years of life. It is indeed known that both diseases may be present simultaneously in the pediatric population, but coexistence may negatively influence the severity of both conditions by increasing the risk of life-threatening asthmatic episodes as well as food-related anaphylaxis. Therefore, an accurate clinical and phenotype characterization of this high-risk group of children with both asthma and food allergy and a more aggressive management might lead to reducing related morbidity and mortality. The aim of this review is to provide an updated overview on the close link between food allergy and asthma and their negative mutual influence.

## 1. Introduction

The strong association between asthma and food allergy is well recognized, but their exact interaction and the underlying mechanisms are still under investigation.

Over the past few decades, an increase in the prevalence of these two atopic conditions has been observed in the pediatric population with a variability reported from 3.5% to 8% of children having food allergies [1,2,3] and about 14% of children experiencing asthma [4].

It is known that 48% of asthmatic patients have food allergy [5] and about half of food allergic children have allergic reactions with respiratory symptoms [6].

The frequency of food sensitization for egg, milk, soy, peanut, wheat, and fish is higher in asthmatics compared to non-asthmatic patients [7,8]; 45% of asthmatics are sensitized to at least one of these foods [7]. On the other hand, patients with multiple and severe food allergies often present asthma often associated with poor control [9,10].

Notably, the coexistence of asthma and food allergy is of significant clinical relevance as it increases the risk of life-threatening asthmatic episodes as well as both food allergen triggered asthmatic episodes or food-related anaphylaxis. It is therefore necessary for clinicians to thoroughly investigate the simultaneous presence of both conditions in order to provide patients with the correct dietary indications and treatments (i.e., inhaled beta-agonists and self-injectable adrenaline devices) for potentially life-threatening events.

This paper reviewed the most important and recent publications concerning the close link between food allergy and asthma and their mutual influence. 

## 2. Methods

The most relevant publications were selected by the authors by employing the PubMed database. On this website, articles were selected ranging from 1 January 1980 to June 2019. The search terms used included the following text words: “food allergy”, “asthma”, “atopic march”, “anaphylaxis”, “children”, “management”, and “prevention”. A restriction for English language was applied. To identify papers for inclusion in our literature review, they were critically examined by title, abstract, or full text. We included all randomized clinical trials, retrospective studies, and literature reviews published in peer-reviewed journals. In addition, to identify further interesting studies, the reference lists of all selected articles were examined.

## 3. Atopic March: The Skin Gateway?

The considerable global increase in the prevalence and severity of atopic diseases including asthma, eczema, allergic rhinitis, and food allergy is a significant public health problem. The presence of one allergic condition increases the risk of developing another, configuring the concept of the atopic march. It describes the natural history of the progression of atopic disease throughout childhood and adolescence.

Classically, atopic dermatitis is considered the gateway to the atopic march that progresses to IgE-mediated food allergy, asthma, and allergic rhinitis [11].

Food specific IgE allergic sensitization reaches a prevalence of 10% at one year of life and can occur early, even before the oral intake of the food itself [12].

In fact, numerous studies suggest early transcutaneous sensitization to food allergens in susceptible individuals. Importantly, loss-of-function mutations in the filaggrin gene, a fundamental structural protein, and mutations in the most recently discovered SPINK5 and corneodesmosin gene [13] generate the disruption of the skin barrier. At the same time, the presence of other genetic and environmental predisposing factors are required for the development of type-2 skin inflammation and progression of atopic march.

In particular, polymorphisms in the thymic stromal lymphopoietin (TSLP) gene increase the risk of atopic dermatitis, food allergy, and asthma [14,15,16], whereas polymorphisms in the interleukin (IL) 33 gene predispose patients to atopic dermatitis and promote the subsequent development of asthma [17,18].

The alteration of skin integrity can be caused not only by a genetic cause, but also by chemical and mechanical factors. Some protease allergens contained in peanut, mites, insects, fungi, and some pollen allow dermal exposure to other allergens for which the individual develops specific IgE sensitization [19].

Atopic dermatitis inflammation results from increased production of IL-25, IL-33, and thymic stromal lymphoprotein, which stimulate the production of IL4, IL5, and IL 13 by innate lymphoid cells and basophilic. In this way, the dendritic cells are activated and can process the allergen by draining into the lymph nodes. Through the interaction with the naive T and B cells, they stimulate a specific TH2 allergen response with the consequent systemic effects in distant tissue sites [20,21,22].

McAleer et al. reported that the risk of developing asthma and allergic rhinitis was higher in children with severe atopic dermatitis and that asthma control was poorer in those with mutations in the filaggrin gene [23]. These data underline the importance of skin integrity in minimizing the possibility of transdermal allergic sensitization for food allergens, particularly for peanuts [24,25]. In studies conducted on mice [26], it seems that the early intake of a food can induce tolerance, while contact between the inflamed skin and the allergen can induce sensitization and consequent allergic reaction after ingestion of the offending food (“double exposure” hypothesis) [27]. For example, the risk of food allergy to peanuts in a child depends on the consumption of the same by the family as well as the amount of this food detectable on children’s toys [24,28]. Certain foods such as peanuts or shrimps may cause hypersensitization through inhalation in subjects who have never eaten them [29].

Some studies have shown that food allergy can be a risk factor for the development of asthma regardless of the co-existing eczema. It has been shown that sensitization for food or inhalant allergens is associated with the onset of school-age asthma, even in the absence of a history of eczema in the first year of life [30].

Subjects with food allergies have a higher risk of developing asthma and rhinitis as shown by a retrospective birth cohort study that emphasized that particular major food allergy such as peanut, milk, and egg may play a more important role in predisposition to asthma (ORs = 1.74, 1.38, and 1.60, respectively) and rhinitis (ORs = 2.59, 1.46, and 1.80, respectively) [11].

In addition, a large meta-analysis highlighted that multiple food allergies and early food sensitization further increased asthmatic susceptibility [31].

An in-depth understanding of the genetic and environmental factors that underlie atopic march as well as the predisposition to develop a TH2 immunological mechanism will allow us to develop new therapeutic approaches in clinical practice.

## 4. Asthma and Food Allergy: What Link?

Food-induced allergic disorders are adverse immunologic potentially life-threatening reactions that occur on exposure to a food. They are categorized into two types: Immunoglobulin-E (IgE) mediated and non-IgE-mediated mechanisms. In the first type, the onset of symptoms occurs rapidly within the first two hours after ingestion and there may be a respiratory, cutaneous, gastrointestinal, and cardiovascular involvement. In the second type, the symptoms are later (12–24 hours after ingestion) and affect mainly the skin and the gastrointestinal tract [5].

Asthma is a common chronic inflammatory disease of the airways characterized by variable and reversible airflow obstruction. Symptoms include episodes of wheezing and shortness of breath, coughing and chest tightness. This condition as well as food allergy is thought to be caused by a combination of genetic and environmental factors. It may also be classified as atopic or non-atopic, even if an allergic component is increasingly recognized in the etiopathogenesis of this complex disease. Some known triggers of asthma attacks include exercise, smoking, sinusitis, medications, food, and food additives. It is well established that inhalant allergen exposure is the most important factor in asthma exacerbation like tree, grass, and weed pollens, mold, animal dander, dust mites, and cockroach droppings.

It is now known that both diseases, asthma and food allergy, may be present simultaneously in the pediatric population, but coexistence may negatively influence the severity of both conditions.

A recently performed meta-analysis resulting from four cross-sectional prevalence studies showed a strong association between food allergy and asthma in the pediatric population, with a reported overall OR = 2.87 [95% CI: 2.05–4.00], proving that food allergy increased the risk of developing allergic asthma. This is even more likely if sensitization to food allergens occurs early within the first few years of life [32,33].

This evidence has been demonstrated by Illi et al., who showed that asthma in school age was more likely in children with an early sensitization to food allergens within the first two years of life, even without sensitization to aeroallergens [34].

Schroeder A et al. reported on the age of early asthma onset and increased asthma prevalence in children with food allergy compared to children without food allergies [9].

Furthermore, the simultaneous allergic sensitization to food allergens and air allergens increased the risk of developing respiratory allergy when compared to a single sensitization, as reported in a recent study by Alduraywish et al. [35].

The pathogenetic mechanism by which food allergy increases the risk of asthma appears to be IgE mediated. Following the exposure to the culprit food, specific IgE determines the production of histamine and leukotrienes by the mast cells, causing inflammation of the airways and subsequent bronchospasm [36].

The most common foods significantly associated with increased risk of developing asthma are peanut, milk, and egg.

A demonstration of the importance of food allergy as a predictor of asthma is the modified asthma predictive index (API). It incorporates IgE mediated sensitization for milk, egg, and peanuts as a secondary risk factor for asthma onset in young children [37].

A minor association was also observed between asthma and non-IgE mediated food allergy. While about half of children with IgE mediated food allergy have asthma, this condition coexists in less than one third of children with non-IgE mediated food allergy [38].

In a British cohort of nearly 300 children with non-IgE median food allergy, about 32% had asthma. Similarly, in an 8-year follow-up study of 89 children with eosinophilic esophagitis, Assa’ad et al. reported an associated asthma condition in 39% of cases [39].

## 5. Food-Triggered Asthma: How to Explain It?

Food allergies can cause mild to severe life-threatening reactions and rarely cause bronchospasm without other symptoms. Cough, rhinitis, and laryngeal edema are the other clinical respiratory manifestations of food allergy [1,6,40].

Isolated food-triggered asthma is a rare event but seems to be more probable in children with eczema and high levels of IgE [41]. Even with the limitations of a small number of enrolled patients and the disparity in the methods of the various studies, the literature shows that about 2–9% of asthmatic children have bronchospasm induced by oral food challenge tests (OFCs) [41,42,43,44].

Even during oral food challenges, gastrointestinal and cutaneous reactions are more frequent than an isolated asthmatic attack [44], but asthma is the most frequent cause of death during an anaphylactic reaction induced by the offending food [45].

It has also been shown that 27% of asthmatic children with positive double-blind, placebo-controlled food challenge (DBPCFC) have a positive methacoline challenge test, despite the absence of wheezing [44].

Although a rare event, asthma can be triggered by exposure to the culprit food allergen in sensitized children, and ingestion or inhalation are the two ways of access for the offending food. To date, the exact mechanism underlying asthma caused by food allergy still remains not perfectly clear [6].

It has been hypothesized that ingested food particles could be inhaled in the airways where an inflammatory cascade is triggered. Both asthmatic and non-asthmatic patients may have an asthmatic attack following exposure to the offending food [1,5].

Aerosolized food proteins such as soy or fish proteins can be responsible for severe, and sometimes fatal, asthmatic attacks [46,47].

Occupational baker’s asthma is due to the inhalation of cereal flour proteins that determine an IgE-mediated reaction at the bronchial level with consequent bronchospasm [48].

A subject can also tolerate an ingested food but also present serious reactions even after inhaling the same food as evidence of a possible modification of allergenicity by digestion [49].

The only therapeutic option in this type of condition is the avoidance of the implicated food. It has been shown that an elimination diet allows the control of asthma in 54.5% of children with food allergy confirmed by DBPCFC [44].

Food elimination must take place not only from the diet, but also from the environment in which the child lives. Asthmatic crises and the use of anti-asthmatic drugs decrease when the offending foods are no longer even cooked in the domestic environment, as evidenced by Roberts et al. in a study where bronchial challenges were performed with aerosolized foods (fish, milk, eggs, chickpeas, buckwheat) in asthmatic, food-allergic children [50].

Exercise may also be responsible for anaphylactic (food-dependent exercise-induced anaphylaxis (FDEIA)) reaction [51] after ingesting the specific offending food or non-specific meal. Asthma symptoms such as wheezing, coughing, and chest tightness often occur in this type of reaction [52].

Furthermore, it is important to underline the clinical impact of several allergic syndromes due to cross-reactivity between aeroallergens and food allergens. Cross-reactivity is an immune-mediated mechanism based on the binding of an IgE antibody to similar allergenic molecules (homologues). Important allergens involved in cross-reactivity are members of pan-allergen families such as PR-10 (pathogenesis-related protein 10), profilins, polcalcins, non-specific lipid transfer proteins, and tropomyosins. A mechanism of cross-reaction may be responsible in subjects primarily sensitized to airborne allergens to the development of allergic symptoms after the ingestion of particular foods. Symptoms related to this cross reactivity can range from oral allergy syndrome to severe anaphylaxis.

These clinical entities are much more frequent in older children, adolescents, and adults in whom up to 60% of IgE-mediated food allergies is associated with an inhalant allergy. In particular, the high prevalence of pollen allergies is related to the increase in the so-called pollen-related food allergies [53]. 

Among patients suffering from pollen-induced allergy, pollen-food syndromes are very common (i.e., cross reactions between tree pollen and apple, cherry, nectarine, peach, hazelnut, carrot, celeriac, soybean, peanut, potato, kiwifruit, and between mugwort pollen and spices, carrot, celeriac, lychee, mango, sunflower seeds, grapes, and peach), while other type of cross-reactions are less common such as house dust mites and shellfish and molluscs, animal epidermis and cow’s milk, and meat and Alternaria-spinach syndrome [54]. 

Careful anamnesis, together with the knowledge of the different cross-reactive allergens, allows for the understanding of these clinical syndromes.

The pattern of sensitizations to common aeroallergens can be determined with skin prick tests or with specific IgE. In the last few decades, component resolved diagnosis offered an important tool for the identification of sensitization due to cross-reactions between aeroallergens and food allergens. Oral provocation testing is still now the gold standard for diagnosis, particularly in patients with an unclear clinical history.

## 6. Food-Induced Asthma: The Offending Foods

The eight most common responsible foods for asthma exacerbations include egg, cow’s milk, wheat, peanut, nuts, fish, soy, and shellfish [44,55,56,57], which are also the allergens responsible for about 90% of food-induced allergies.

Studies of children with food allergic sensitization highlight that an early hen’s egg allergy, especially with coexisting eczema, could be a predictor for respiratory allergic disease during childhood. In a birth cohort of 1218 children, Tariq SM et al. found that egg allergy in infancy predicted asthma, rhinitis, and allergic disease at four years of age, with a positive predictive value (PPV) of 80% and 55.0% in children with and without coexistent eczema, respectively [58].

Rhodes HL et al. studied early life risk factors for adult asthma in a birth cohort of one hundred babies of atopic parents: skin sensitivity to egg and cow’s milk, or both in the first year was independently predictive of adult asthma (OR = 10.7; 95% CI: 2.1–55.1; *p* = 0.001; sensitivity = 57%; specificity = 89%) [59].

## 7. Asthma and Food Allergy: The Combined Effects

With regard to the close association between asthma and food allergy, several findings suggest that their coexistence may increase the severity of both conditions.

Recently food allergy, in addition to sensitization for inhalants, blood increase levels of eosinophils and basophils, has been identified as a distinctive feature of severe asthma [60].

In fact, food allergy is a significant risk factor for life-threatening asthmatic exacerbations and pediatric intensive care unit admission for asthmatic children. Roberts G et al. performed a case-controlled study on children (1–16 years) ventilated for an exacerbation and demonstrated that food allergy, together with poorly controlled asthma, were independently associated with these life-threatening events [61].

Friedlander JL et al. prospectively surveyed 300 asthmatic children of whom 24% had food allergy and 12% multiple food allergies. These patients had an increased risk of hospitalization (OR = 2.35; 95% CI: 1.30–4.24; *p* = 0.005), and use of controller medication (OR = 1.99; 95% CI: 1.06–3.74; *p* = 0.03). These data were even more evident for patients with multiple food allergies [10].

Vogel et al. showed that food allergy was an independent risk factor for potentially fatal childhood asthma in a pediatric population of 72 patients admitted to a pediatric intensive care unit for asthma attack, compared to two control groups of 108 patients admitted to a nursing floor and 108 outpatients [62].

Numerous studies have focused on the specific food allergy as a cause of increased asthma morbidity [63,64].

Simpson et al. evaluated 201 asthmatic children aged three months to 14 years by comparing the group with asthma and food allergy (88 children) with the group without coexisting food allergy. The coexistence of asthma and peanut and milk allergies were both associated with an increased number of hospitalizations (*p* = 0.009, 0.016), and milk allergy with a greater need of systemic steroids (*p* = 0.001) [65].

Furthermore, food allergic polysensitization has been associated with a greater number of hospitalizations and accesses in emergency departments as well as greater use of systemic steroids for major asthma exacerbations [66].

A population-based case-control study on 45 asthmatic patients aged 5 to 50 years who needed ventilation in the intensive care unit showed that cases were more likely to have food allergy (OR = 3.6; 95% CI: 1.6–8.2) and/or have had anaphylaxis (OR = 5.3; 95% CI: 2.7–10.6) when compared to controls treated in the emergency department and in an outpatient setting [67].

As food allergy can make asthma more life-threatening, asthma may negatively influence the severity of food allergy, thus increasing the risk of anaphylaxis. To demonstrate this, Boyano-Martinez et al. conducted a study on 88 children allergic to cow’s milk and concluded that the frequency of more severe reactions was 10 times greater in children with asthmatic comorbidity (OR = 10.2; 95% CI: 1.13–91.54) [68].

Bock SA et al. analyzed 32 fatal cases of anaphylaxis and their careful characterization, based on the available data, has led to recognizing asthma as the common denominator of these fatal events [69].

## 8. Management and Prevention

The management of children with food allergy and asthma is a debated topic. Considering the close association between these two clinical conditions, it is extremely important to investigate the presence of asthma in a child with food allergy and food allergy in a child with asthma.

Detailed anamnesis should be carried out to evaluate the potential correlation between the clinical symptom and the eventual responsible trigger.

Therefore, particularly in the suspicion of food allergy, an accurate identification of the culprit food(s) is essential to allow avoidance.

Like all other pathologies, the diagnosis of adverse food reactions must follow the traditional course of a good medical history, an accurate physical examination, and the support of laboratory tests and in vivo tests such as skin prick tests to the suspected foods and serum-specific IgE antibodies [70]. An oral food challenge (OFC) [70,71] is still now the gold standard to ascertain an allergic reaction, or a food–exercise challenge test to identify children with FDEIA [72].

To date, a more important eosinophilic inflammation in the airways of asthmatic subjects with food allergy has been demonstrated when compared to non-allergic asthmatics, in terms of increased airway hyperactivity, FeNO, and blood eosinophilia. These tests should be performed for a better characterization of those high-risk patients who clearly require a more aggressive therapeutic approach [73,74].

There is considerable scientific interest for the primary prevention of food allergy. The European Academy of Allergy and Clinical Immunology EAACI food allergy primary prevention guideline provides a wide range of antenatal, perinatal, neonatal, and childhood strategies. It recommends a healthy balanced maternal diet during pregnancy and breastfeeding. Exclusion of any foods (including highly allergenic foods) from the maternal diet is not recommended, as this has not been shown to prevent allergies. There is some evidence that omega-3 fatty acids (found in oily fish) during pregnancy and breastfeeding may reduce allergic sensitization to egg. There is no evidence to recommend the intake of any supplements such as probiotics. 

Exclusive breastfeeding is recommended for all infants for the first 4–6 months. If this is not possible, an hypoallergenic formula should be appropriate only for high-risk infants in the first four months of life. For other infants and those after four months of life, a standard cow-based milk formula is recommended. Solid foods should not be introduced before four months, but should not be delayed beyond six months according to the child’s psychomotor development. Regarding food allergy prevention and the timing of ‘highly allergenic’ food introduction (cow’s milk, hen’s egg, and peanuts), this guideline does not justify the recommendations for either withholding or encouraging exposure to these foods after the age of four months regardless of child atopic risk. There is some evidence that the introduction of common allergenic foods should not be delayed, however, there is a need for future studies to clarify the optimal timing of the introduction of each complementary food [75].

Prevention studies on the role of early food avoidance in the development of atopy and asthma are currently unclear. The Isle of Wight Prevention study showed a lower risk of developing asthma at eight years of age, but its role in the prevention of asthma in later years of life has not yet been demonstrated [76,77,78,79].

Regarding the therapeutic treatment of food allergy, the avoidance of the offending food is the cornerstone [80,81].

Furthermore, caregivers, and not only the affected patient, must be careful to avoid accidental ingestion or inhalation exposure [82].

Therefore, as indicated by the EAACI guidelines, the prescription of injectable adrenaline devices to this high-risk group of children with both asthma and food allergy [5,83,84] is recommended to reduce future morbidity and mortality. Patients should be instructed to recognize and promptly treat symptoms as indicated by a provided action plan by use of rescue medications including short-acting beta2-agonists, antihistamine drugs, and self-injectable adrenaline.

Food allergies and severe asthma cause a significant burden to affected patients and their families, resulting not only in dietary restrictions and risk of severe reactions, but also in many disease-related academic and social obstacles [85].

School is a place where children spend many years of their lives for many hours and allergic and asthmatic children may be able to be included and involved in all available school and extra-school activities. For this reason, it is necessary to adopt all environmental and behavioral prevention measures necessary to ensure the possibility of being able to safely attend school throughout the school year. Several precautions are necessary to adapt the school environment to the needs of the allergic child: (1) The activation of all measures of environmental prevention, necessary to guarantee a healthy air quality thanks to the abatement of allergens, indoor and outdoor environmental pollutants; and (2) The activation of procedures and contrast measures aimed to eliminate food contamination with food allergens. Careful control procedures must be implemented throughout the food supply chain from the procurement of raw materials to the delivery of the meal to school and as much as possible during meal consumption and school activities. It is equally necessary to guarantee operational protocols and trained personnel, who are capable of administering drugs in the event of allergic anaphylactic and/or asthma crises at school. 

During school activities, children could be exposed in a non-obvious way to potential allergens hidden in nondietary sources, reporting apparently unexplainable chronic allergic symptoms. Examples are the plasticine that contains wheat, the stuffed animals that can be made with crushed or ground nut shells, finger paint containing wheat, milk, corn and oat, crayons containing soy, dustless chalk containing casein, and balloons manufactured with natural rubber latex [86]. Alternative materials and precautions should be used to avoid potential reactions for allergic children.

## 9. Conclusions

This paper highlights the importance of the association between asthma and food allergy and their mutual influence, even if the mechanisms underlying their coexistence are still not yet clear.

In the pediatric population, IgE-mediated allergic sensitization, particularly for egg, represents a predictor marker for the development of asthma. These data should lead to a greater awareness of how food allergy management, like of other risk factors such as hereditary traits and environmental factors, is pivotal in reducing the risk of developing asthma in children.

The coexistence of asthma and food allergy makes the symptoms potentially more serious.

Although rare, food-induced asthma attack, especially in patients with poor asthmatic control, is the main cause of death in food allergic reactions. A careful evaluation of food triggers and offending food avoidance is a crucial part in the management of this high risk group of patients, together with the provision of a clear action plan regarding the use of emergency drugs. To date, fatal anaphylactic reactions continue to occur, so a careful characterization of this high-risk group of children with both asthma and food allergy might lead to a safer management plan, thus reducing morbidity and mortality.

## Abbreviations

CI	Confidence Interval
DBPCFC	Double-blind, placebo-controlled food challenge
EAACI	European Academy of Allergy and Clinical Immunology
FDEIA	Food-dependent exercise-induced anaphylaxis
FeNO	Fractional exhaled nitric oxide
OR	Odds ratio
ORs	Odds ratios
OFC	Oral food challenge
